# St. Mary’s Infant Asylum and Lying-In Hospital, Buffalo, N. Y.

**Published:** 1902-03

**Authors:** L. G. Hanley

**Affiliations:** Chief Obstetrician


### St. Mary's Infant Asylum and Lying-in Hospital, Buffalo, N. Y.

### Report of Cases for the Year 1901 in Maternity Department.

By L. G. HANLEY, M. D., Chief Obstetrician.

Case I.—Age 32, multipara; uterine pains began at 2 a.m., December 31, 1900; membranes ruptured at 12 p.m. Twins. Double breach. First child, male, born at 11.25; second, female, at 11.45; two sacs, one placenta; no laceration. Weight of first child, 7½ lbs. Weight of second child, 8 lbs. No complications; recovery normal.

Case II.——— H., age 20, primipara; uterine pains began 8 p.m., January 20; membranes ruptured at 2 a.m.; child delivered at 3.15 a.m. Jan. 21. Position of child, first; female. Weight, 7 lbs. No complications.

Case III.—Mrs. B., age 23, multipara. Uterine pains began 12 p.m. Jan. 21. Membrane ruptured 3 a.m. Jan. 22. Child delivered at 6 a.m. Jan. 22; position of child, first; male. Weight, 10 lbs. Complications, cord around neck and child.

Case IV.——— F., Age 15 years. Primipara; uterine pains began at 1 a.m. Jan. 24; membrane ruptured at 2 a.m. Jan. 24; male child delivered 7.30 a.m. Jan. 24. Breach presentation. Sacro-left-anterior; complications—cord around the leg and neck. Incomplete laceration of sphincter muscle. Laceration repaired with silk-worm gut. Dead baby. Patient made good recovery.

Case V.——— C., age 22; primipara; uterine pains began 5 a.m. Feb. 8. Membranes ruptured 6.30 a.m. Feb. 8. Child delivered 7.30. Position of child, first. Female, weight, 7 lbs. No complications.

Case VI.—Mrs. McD., age 32; multipara; uterine pains began at 9 p.m. Feb. 12. Membranes ruptured at 11 a.m. Feb. 13; child delivered at 11.45; female; position of child, first; weight, 8 lbs. No complications.

Case VII.——— S., age 48; primipara; uterine pains began at 7 a.m. Feb. 13. Membranes ruptured at 3 p.m. Child delivered at 11.45 p.m. Position of child, first. Complication, cord around the neck twice. Chloroform, forceps and low operation. Female; weight 7 lbs. Incomplete laceration, repaired with silk-worm gut.

Case VIII.——— D., age 22; primipara; pains began at 6 a.m. Feb. 16; membranes ruptured at 11 p.m. Feb. 17; child delivered at 6 p.m. Feb. 17. Position of child, first. Cord around child's neck twice. Male; weight, 9 lbs. No complications.

Case IX.——— H., age 28; primipara; uterine pains began 11 p.m. Feb. 17; membranes ruptured at 4 a.m. Feb. 18. Child delivered 5.30 a.m. Female, weight, 8 lbs. No complications.

Case X.——— L., age 16; primipara; uterine pains began at 3 p.m.; membranes ruptured at 6 p.m.; child delivered at 7.15 p.m. Chloroform, forceps, low operation. Position of child, first. Female; weight, 7 lbs.

Case XI.——— E., age 28; primipara; uterine pains began at 1 p.m. Feb. 21; membranes ruptured at 6 a.m.; child delivered 3.30 p.m. Position of child, first; forceps, low operation; no chloroform, post partum hemorrhage severe, checked by ergot; Credé's method and hot saline douche; female; weight, 7 lbs.

Case XII.—Mrs. B., age 22; primipara; uterine pains began at 4 a.m. Feb. 24; membranes ruptured at 1.30 p.m. Feb. 25; child delivered 12.30 a.m. Feb. 26. Male; weight, 8½ lbs.; cord around neck and arm.

Case XIII.——— M., age 25; primipara; uterine pains began at 12 p.m. Mar. 4; membranes ruptured at 8 a.m. Mar. 5; child delivered at 9.20 a.m. Position, first. Complications, cord around neck and arm, forceps; no chloroform. Incomplete laceration of perineum; repaired with silk-worm gut; male, weight, 7½ lbs.

Case XIV.——— C., age 23; primipara; uterine pain began at 4 p.m. Mar. 12; membrane ruptured at 4 a.m. Mar. 13; child delivered 7.30 a.m. Mar. 13. Position, first; male, weight, 8 lbs.

Case XV.——— M., age 20; primipara; uterine pains began at 5 a.m. Membranes ruptured at 2.30 p.m. Mar. 16. Child delivered 3.15 p.m. Position, second. Complications, cord around body and neck; female; weight, 8 lbs.

Case XVI.——— D., age 18; primipara; uterine pains began at 8 a.m. Mar. 16. Membranes ruptured at 1 p.m. Mar. 16; child delivered at 3.30 p.m. Mar. 16. Position of child, first; female; weight, 8½ lbs. No complications.

Case XVII.——— R., age 21; primipara; uterine pains began 11 p.m. Mar. 22; membranes ruptured at 2 a.m. Mar. 23; child delivered 6 a.m. Mar. 23. Forceps, low operation; no chloroform. Cord around neck; laceration through sphincter, repaired with silk-worm gut. Male; weight, 9 lbs.

Case XVIII.——— S., age 23; primipara; uterine pains began at 8 a.m. Mar. 27; membranes ruptured at 6 p.m. Mar. 27. Child delivered at 8 p.m. Position, first; cord around neck twice; male; weight, 8 lbs.

Case XIX.—Mrs., age 27; multipara; uterine pains began 3 p.m. Mar. 28; membranes ruptured at 5 p.m.; child delivered at

12 p.m. Mar. 28; position, first; cord around neck and arm; female; weight, 9 lbs.

Case XX.—Mrs. S., age 20; multipara; uterine pains began at 5 a.m. Mar. 29; membranes ruptured at 9 p.m.; child delivered at 11.30 p.m. Position, first; female; weight, 10 lbs.

Case XXI.——— L., age 20; multipara; uterine pains began at 4 p.m. April 3; membranes ruptured at 1 a.m. April 4; child delivered at 1.45 a.m.; position, first. Cord around the neck; male; weight, 7 lbs.

Case XXII.——— J., age 18; primipara; uterine pains began at 2 p.m. April 22; membranes ruptured at 7 a.m. April 23; child delivered at 7.45 a.m. Position, first. No complications; weight, 7½ lbs; male.

Case XXIII.——— F., age 18; primipara; uterine pains began 12 a.m. April 19; membranes ruptured 7 p.m.; April 9; child delivered at 8.45 p.m. Position, first; adherent placenta; female; weight, 6½ lbs.

Case XXIV.——— E., age 24; multipara; uterine pains began at 4 a.m. April 25; child delivered 11.45 a.m. April 25. Forceps, chloroform, high operation; face presentation with cord around the neck; dry labor; male; weight, 8 lbs.

Case XXV.——— H., age 22; primipara; uterine pains began at 2 a.m. May 12; membranes ruptured 4 a.m. May 12; child delivered at 10.15 a.m. May 12. First position; chloroform, forceps delivery, low operation; cord around neck twice and around body once; male; weight 8 lbs.

Case XXVI.——— B., age 30; primipara; puerperal eclampsia; was admitted to hospital at 10 p.m. April 8, in labor. Slight dilatation of os. Patient has had several convulsions; catheterised, and urine showed about 90 per cent. albumin. Steam bath given; also croton oil; veratrum viridi. The uterus was empty three and a half hours after admission; female; weight, 7 lbs.; patient died at 10 p.m. April 19, during a convulsion. Steam baths, croton oil, saline injections were used.

Case XXVII.—Mrs. H., age 39; multipara; uterine pains began at 1 a.m. May 14; membranes ruptured at 1 p.m. May 15; child delivered at 2.45 p.m.; chloroform, forceps, low operation; position, first; male; weight, 7 lbs. No complications.

Case XXVIII.——— C., age 18; primipara; uterine pains began 9 p.m. May 18; membranes ruptured at 10 a.m. May 19; child delivered at 11.30 a.m. May 19; position, first; female; weight, 8½ lbs.

Case XIX.——— B., age 20; multipara; uterine pains began at 7 a.m. May 21; membranes ruptured at 1 p.m. May 22; child delivered at 2.15 p.m. First position; no complications; female; weight, 7½ lbs.

Case XXX.—Mrs. T.; age 20; primipara; uterine pains began at 6 p.m. May 21; membranes ruptured at 6.15 p.m. Cord around child and neck four times. Position, second; female; weight, 6 lbs.

Case XXXI.——— A., age 20; primipara; uterine pains began at 4 p.m. June 7; membranes ruptured at 12 m. Child delivered at 1.30 p.m. June 8. Position, first; complications, cord around neck and arm; chloroform, forceps; male; weight, 8 lbs.

Case XXXII.—Mrs. R., age 21; multipara; uterine pains began at 12 p.m. June 20; child delivered at 2.15 a.m. June 21; female; weight, 6½ lbs. Face presentations; roomy pelvis.

Case XXXIII.——— N., age 18; primipara; uterine pains began at 1 p.m. May 26; membranes ruptured at 10 p.m. May 27; child delivered at 11.30 p.m. May 27. Position of child, second. Post partum hemorrhage; female; weight, 7½ lbs.

Case XXXIV.——— J., age 17; uterine pains 9 p.m. June 27; primipara; membranes ruptured at 1 a.m. June 28; child delivered at 1.45 a.m. June 28. Position, first; cord around neck and arm; male; weight, 8 lbs.

Case XXXV.——— S., age 21; primipara; uterine pains began 6 p.m. June 28; membrane ruptured at 1.30 a.m. June 29; child delivered at 2.15 a.m. Position, first; cord around neck; female; weight, 7½ lbs.

Case XXXVI.—Mrs. L., age 31; multipara; patient admitted to hospital when she was four and a half months pregnant; she was suffering from tuberculosis; extremely emaciated, and a cavity in either lung. She was delivered of a 3½ lb. baby, male, when 8 months pregnant. She died one day after confinement. Labor was normal.

Case XXXVII.——— F., age 24; primipara; uterine pains began at 10 a.m. July 13; membranes ruptured at 12.30 p.m. July 14; child delivered at 2.30 p.m. July 14; position of child, second. Chloroform, forceps, low operation; female; weight, 8½ lbs.

Case XXXVIII.——— M., age 16; primipara; uterine pains began at 1 a.m. July 16. Membranes ruptured at 6 a.m.; child delivered at 10 a.m. July 16; position, first; normal labor; male; weight, 9 lbs.

Case XXXIX.——— A., age 17; primipara; uterine pains began at 1 a.m. July 17; membranes ruptured at 3 a.m. July 17; child delivered at 10.30 a.m. July 17; chloroform, forceps, low operation, adherent placenta; female; weight, 7½ lbs.

Case XL.——— L., age 39; primipara; uterine pains began at 12 p.m. July 25; membranes ruptured at 2 a.m. July 27; child delivered at 1.30 p.m. July 27; chloroform, forceps, high operation. Position, fourth; female; weight, 7 lbs. Incomplete laceration.

Case XLI.——— M., age 17; primipara; uterine pains began at 12 m, July 28; membranes ruptured at 5 a.m.; child delivered at 4.45 a.m. July 29. Normal labor; position, first; male; weight, 8 lbs.

Case XLII.——— A., age 17; primipara; uterine pains began 2 a.m. July 29; membranes ruptured at 4.30 a.m. July 29; child delivered at 12.45 p.m.; chloroform, forceps, low operation; cord around neck; male; weight, 8½ lbs.

Case XLIII.—Mrs. B., age 27; multipara; uterine pains began 9 p.m. Aug. 6. Membrane ruptured 4 a.m. Aug. 7; child delivered 9 a.m. Aug. 7; position, first; male; weight, 8 lbs.

Case XLIV.——— J., age 22; primipara; uterine pains began at 6 p.m. Aug. 17; membranes ruptured 7 a.m. Aug. 18; child delivered 9 a.m., Aug. 18. Forceps delivery, low operation; position, third; male; weight, 7 lbs.

Case XLV.—Mrs. H., age 23; multipara; uterine pains began at 10 a.m. Aug. 23; child delivered 6.30 p.m. Aug. 23. Dry labor; position, second; forceps; female; weight, 8 lbs.

Case XLVI.——— B., age 22; primipara; uterine pains began at 9 a.m. Aug. 25; membrane ruptured at 8 a.m. Aug. 25. Chloroform, forceps delivery. Position, first; female; weight, 7 lbs.

Case XLVII.—Mrs. A., age 23; multipara; uterine pains began at 10 a.m. Aug. 23; membranes ruptured at 3 p.m. Aug. 23; child delivered at 12.45 p.m. Aug. 26. This patient was brought to the hospital by Dr. C. who said that she had been in labor for three days, under the care of a midwife. Position transverse. Scapular, right posterior. Arm presented. Midwife had made several efforts to deliver by using traction on arm. Arm is black and in a gangrenous condition, from constriction of the os. I chloroformed patient, rendering the arm and vagina as aseptic as possible, and performed version. Arm was in a state of decomposition. Male; weight, 6 lbs.; patient left hospital in nine days. Would not remain, as she expressed it she was perfectly well.

Case XLVIII.——— R., age 24; primipara; uterine pains began at 12 p.m. Sept. 2; membranes ruptured at 10 a.m. Sept. 3. Child delivered at 11.45 a.m. Sept. 3. Position of child, first; chloroform, forceps delivery, low operation. Female; weight, 8 lbs.

Case XLIX.——— B., age 24; primipara; uterine pains began at 4 p.m. Sept. 6; membrane ruptured at 11.15 p.m. Sept. 6; child delivered at 12.15 p.m. Sept. 7. First position; chloroform, forceps, low operation; female; weight, 6½ lbs.

Case L.——— M., age 18; primipara; uterine pains at 5.45 p.m. Sept. 6; membranes ruptured at 11.30 p.m. Sept. 6. Child delivered at 1.00 a.m. Sept. 7. Position of child, first. Cord around neck; male; weight, 9 lbs.

Case LI.——— N., age 28; primipara; uterine pains began at 2.00 a.m. Sept 7. Membrane ruptured at 8.30 p.m.; child delivered at 10.30 p.m. Chloroform, forceps delivery, low operation; female; weight, 8 lbs.

Case LII.——— H., age 25; primipara; uterine pains began at 8.00 a.m. Sept. 14; membranes ruptured at 6.00 p.m. Sept. 14; child delivered at 8.30 p.m. Sept. 14. First position; cord around the neck; male, weight, 7½ lbs.

Case LIII.——— B., age 22; primipara; uterine pains began at 6 p.m. Sept. 23. Membranes ruptured at 11 p.m. Sept. 23. Child delivered at 12.45 a.m. Sept. 24. Position of child, first; female; weight, 7½ lbs.

Case LIV.——— N., age 21; primipara; uterine pains began at 12 p.m. Sept. 30; membranes ruptured at 3.00 a.m. Oct. 1; child delivered at 6 a.m. Oct. 1. Complications, cord around the neck; male; weight, 7½ lbs.

Case LV.——— E., age 18; primipara; uterine pains began at 9 p.m. Oct. 3; premature birth; five months; breach; male.

Case LVI.——— R., age 18; primipara; uterine pains began at 9 p.m. Oct. 19; membranes ruptured at 2 a.m. Oct. 19. Child delivered at 3.15 a.m. Chloroform, forceps, low operation; adherent placenta; incomplete laceration of perineum, repaired with silk-worm gut; position, first; female; weight, 7½ pounds.

Case LVII.— Mrs. W., age 22; primipara; uterine pains began at 4 a.m. Oct. 21; child delivered at 2.30 a.m. Oct. 22; chloroform, forceps delivery; face presentation M.L.A.; female, weight, 8 lbs.

Case LVIII.——— M., age 32; primipara; uterine pains began at 4 a.m. Oct. 30; membranes ruptured at 7.00 a.m. Oct. 30. Child delivered at 3.30 p.m. First, position; chloroform, forceps; cord around the neck; female; weight, 8 lbs.

Case LIX.——— M., age 26; primipara; uterine pains began at 7 a.m. Oct. 9. Membrane ruptured at 11.30 p.m. Oct. 9; child delivered at 12.15 a.m. Oct. 10. Slight laceration of perineum, repaired with silk-worm gut; position, second; male; weight, 7 lbs.

Case LX.——— L., age 19; primipara; uterine pains began at 5 p.m. Nov. 6th; child delivered at 11 p.m. Nov. 7; position of child, first. Adherent placenta; female; weight, 6½ lbs.

Case LXI.—Mrs. H., age 31; multipara; uterine pains began at 5 p.m. Nov. 13. Membranes ruptured at 1 a.m. Nov. 14.

Child delivered a 2.15 a.m. Nov. 14. Chloroform, forceps, low operation. Position, third; female; weight, 8 lbs.

Case LXII.——— A., age 28; primipara; uterine pains began at 3 p.m. Nov. 18; membranes ruptured at 9.15 a.m. Nov. 19; child delivered at 10.15 a.m. Nov. 19. Position of child, first. Chloroform, forceps, low operation, cord around neck; female; weight, 11 lbs.

Case LXIII.——— M., age 19; primipara; uterine pains began at 7.00 a.m. Nov. 21; membrane ruptured at 3.00 a.m. Nov. 22; child delivered at 1.15 Nov. 22. Position, second; chloroform, forceps; cord around the neck and arm. This case was a monstrosity; anacephalic; female; weight, 7½ lbs.

Case LXIV.——— M., age 31; primipara; uterine pains began at 12 M. Nov. 24. Membranes ruptured at 2.30 p.m. Nov. 24. Child delivered at 8.15 p.m. Cord around child and neck; male; weight, 7½ lbs.

Case LXV.——— K., age 24; primipara; uterine pains began at 11.00 p.m. Nov. 25; membrane ruptured at 11.30 a.m. Nov. 26; child delivered at 1.15, Nov. 26; position of child, first. Female; weight, 6½ lbs.

Case LXVI.——— L., age 23; primipara; uterine pains began at 10.00 p.m. Nov. 25; membranes ruptured at 2.00 p.m. Nov. 6. Child delivered at 5.45 p.m. Nov. 6; position of child first; complications, cord around the neck; male; weight, 8 lbs.

Case LXVII.——— H., age 17; primipara; uterine pains began at 8 a.m. Dec. 6. Membrane ruptured at 6.30 p.m. Nov. 6; position. first; female; weight, 6 lbs.

Case LXVIII.——— A., age 45; primipara; membrane ruptured Dec. 6; child delivered at 5.30 p.m., Dec. 7. Position of child, first; complication, cord around neck and arm. Incomplete laceration; female; weight, 7 lbs.

Case LXIX.——— L., age 20; primipara; uterine pains began 5.00 p.m. Dec. 17. Membrane ruptured 1.00 a.m. Dec. 18; child delivered 3.00 a.m. Dec. 18; position, second. Chloroform, forceps; male; weight, 8½ lbs.

Case LXX.——— M., age 23; primipara; uterine pains began at 8.00 p.m. Dec. 23; Membrane ruptured at 1.30 a.m. Dec. 24; child delivered at 2.15 a.m. Dec. 24; position of child, first. Cord around neck and arm; female; weight, 7 lbs.

On service, Drs. J. Stoddart, P. J. Candee, C. J. Carr, Wm. J. O'Donnell; nurse, Miss B. Fitzgerald.

428 Porter Avenue.

